# Sharing research results with Latina breast cancer survivors who participated in a community-engaged behavioral RCT study: a descriptive cross-sectional survey study

**DOI:** 10.1186/s13063-021-05945-8

**Published:** 2022-01-08

**Authors:** Jackie Bonilla, Alia Alhomsi, Jasmine Santoyo-Olsson, Anita L. Stewart, Carmen Ortiz, Cathy Samayoa, Alma Torres-Nguyen, Helen Palomino, La Verne Coleman, Aday Urias, Nayeli Gonzalez, Silvia Araceli Cervantes, Ysabel Duron, Anna María Nápoles

**Affiliations:** 1 National Institute on Minority Health and Health Disparities, National Institutes of Health, 9000 Rockville Pike, Building 3, Floor 5, Room E08, Bethesda, MD 20892 USA; 2 Division of General Internal Medicine, Department of Medicine, University of California San Francisco (UCSF), 3333 California St., Suite 335, San Francisco, CA 94143-0856 USA; 3 University of California San Francisco, Institute for Health & Aging, Center for Aging in Diverse Communities, 490 Illinois Street, 12232, San Francisco, CA 94158 USA; 4 Círculo de Vida Cancer Support and Resource Center, 2601 Mission Street, Suite 702, San Francisco, CA 94110 USA; 5 Health Equity Research Lab, Department of Biology, San Francisco State University, 1600 Holloway Ave, San Francisco, CA 94132 USA; 6 Kaweah Delta Health Care District, 400 W. Mineral King, Visalia, CA 93291 USA; 7 Cancer Resource Center of the Desert, 444 So. 8th St, Suite B-3, El Centro, CA 92243 USA; 8 WomenCARE/Entre Nosotras, Family Service Agency of the Central Coast, 2901 Park Avenue, Suite A1, Soquel, CA 95073 USA; 9 Latino Cancer Institute, 123 East San Carlos Street, #413, San Jose, CA 95112 USA

**Keywords:** Community-based participatory research, Cancer survivors, Hispanic Americans, Information dissemination, Minority groups, Rural population, Community participation, Surveys and questionnaires, Language, Literacy

## Abstract

**Background:**

An often heard and justifiable concern of ethnic minorities is related to researchers’ lack of attention to sharing the results of a study with participants after the study has concluded. Few studies have examined the effects of returning overall study results on participants’ attitudes, especially among populations underrepresented in research. Among Latina research participants, providing a summary of study results could enhance participation in research. We assess Latina breast cancer survivors’ reactions to receiving study results and their attitudes about participating in future studies.

**Methods:**

For this cross-sectional survey study, all women who had participated in two behavioral randomized controlled trials (RCTs) were mailed a letter summarizing the study results (using written and graphic formats) and a questionnaire assessing problems and understanding the results, importance of sharing results, willingness to participate in future studies, and format preferences for receiving the results. A postage-paid envelope for returning the completed questionnaire was included. Logistic regression examined the associations of age, education, and rural/urban residence on format preferences and willingness to participate. The survey sample consisted of 304 low-income, predominantly Spanish-speaking Latina breast cancer survivors (151 from urban and 153 from rural communities) who had participated in two RCTs testing a stress management program designed for Latina breast cancer survivors.

**Results:**

Ninety-two women returned the questionnaires (30.3%). Most of the women (91.1%) indicated that they had no trouble understanding the results of the study, and 97% agreed that it is very/extremely important for researchers to share the study result with the participants. The majority (60.2%) reported that receiving the results increased their willingness to participate in future studies. About half (51.7%) did not have a format preference, 37.4% preferred written summaries, and 10.9% preferred graphs.

**Conclusions:**

This study is an important first step to understanding the impact of returning study results among a population that is underrepresented in research. Returning the results of studies and understanding the impact of doing so is consistent with maintaining community involvement in all phases of research. The findings suggest that sharing aggregate research results in simple language yields few problems in participants’ understanding of the results and is viewed as important by participants.

**Trial registration:**

ClinicalTrials.govNCT02931552 Date registered: October 13, 2016 and NCT01383174 Date registered: June 28, 2011.

**Supplementary Information:**

The online version contains supplementary material available at 10.1186/s13063-021-05945-8.

## Background

Due to historical and structural factors in the USA, ethnic minorities tend to report greater mistrust of researchers [1, 2]. This perspective is so pervasive that scholars of community-based participatory research (CBPR) have coined the phrase “helicopter research” to describe researchers who drop in, collect data, and do not return to the community [3]. The lack of communication of the results could further exacerbate feelings of mistrust and make minority participants less likely to participate in future studies [3]. The magnitude of this issue is evidenced in the mixed response among ethnic minorities to participating in the coronavirus infectious disease 19 (COVID-19) clinical trials despite being disproportionately burdened by the pandemic [4, 5]. Furthermore, COVID-19-related disparities have illustrated the need for community engagement in all stages of research, including dissemination and evaluation [6].

An important tenet of CBPR research is to engage communities as equal partners in all phases of the research, including dissemination of results [7], and participants overwhelmingly prefer that research findings be shared with them [8, 9]. A national survey of ethnically diverse persons enrolled in research volunteer registries found that about three-quarters of all participants valued receiving research results and thus would be more likely to trust researchers and participate in research [10]. Furthermore, in this study, African Americans and Latinos were more likely than Whites to indicate that receiving research results would increase their trust in researchers [10]. Providing results to study participants demonstrates respect for the participants as collaborators, makes them feel valued and included after the research has ended, and potentially facilitates their willingness to participate in future projects [8, 9, 11, 12].

Studies examining the return of results have been conducted in the context of returning genomic results [13, 14]; however, empirical data on optimal methods for return of other types of results is lacking [15]. Critically, more research needs to be done on sharing results in studies testing behavioral interventions in minority communities due to their underrepresentation in health research [16]. In a study examining health research participant preferences, only about 33% of participants stated that they had received results in the past [17], and rates of returning results are low, even in CBPR studies [18]. Clear gaps in the literature exist in terms of data on the preferences of research participants for return of results, the best channels and formats for returning results, and the effects of returning results of studies among the general population [17], as well as populations that are underrepresented in research such as Latinos. Dissemination of the study results to the participants is a step in the research process that is almost exclusively ignored and rarely studied, especially among underserved populations who also tend to be underrepresented in research.

The objective of this study was to better understand the reactions to receiving study results of Spanish-speaking Latina breast cancer survivors who had participated in two RCT studies testing a culturally tailored stress management program to improve quality of life. In this descriptive cross-sectional survey study, aggregate results from the RCT in which they had participated were shared through a letter mailed to the participants using two presentation formats. We report here the results of a brief survey that was included with the mailing of the results that asked women about their reactions to receiving the results.

Because women in both study samples were from low-income, limited English-speaking rural and urban communities, we aimed in this descriptive study to assess whether (1) women read and understood the study findings, (2) if they preferred a written or graphical presentation of findings, (3) how important they felt that returning the results was to them, and (4) whether receiving the results affected their willingness to participate in future studies. Also, due to the vulnerable nature of the sample, in exploratory analyses, we examined whether women’s understanding of the results, willingness to participate in future studies, or format preference (graphic or written) varied by age, education, or rural/urban residence.

## Methods

**Fig. 1:**
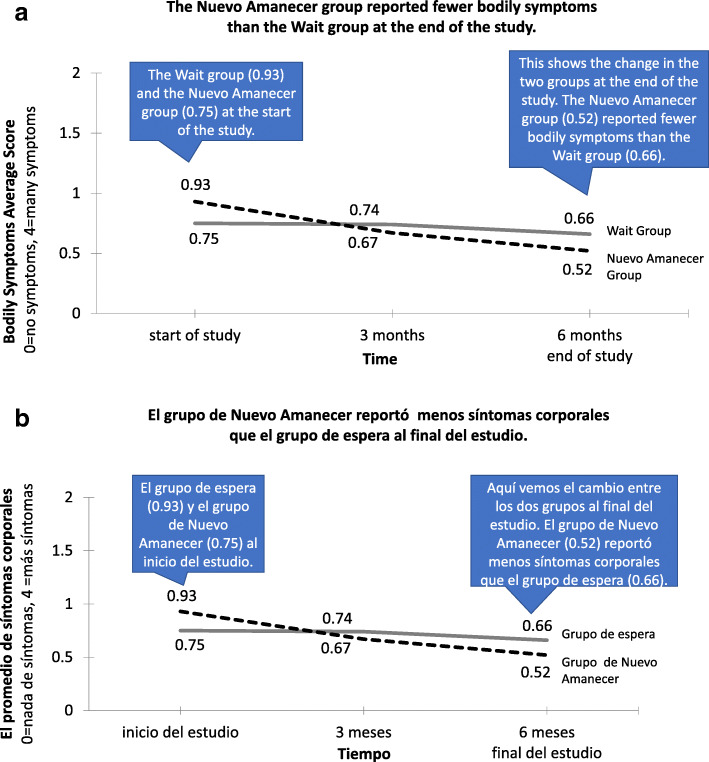
Results in English and Spanish

The sample for this descriptive cross-sectional study consisted of all 304 (151 urban and 153 rural) Spanish-speaking Latina breast cancer survivors who had participated in two RCTs of Nuevo Amanecer, an 8–10-week cognitive-behavioral stress management program. Within about 6 months after the completion of each RCT study, all 304 RCT study participants were sent a mailing that included a letter in Spanish (all were native Spanish-speakers) thanking them for their participation and providing the results of the RCT in two simple formats: a plain language, bulleted list of the main findings (Fig. S1: English and Spanish versions of the letter with aggregate RCT results sent to Nuevo Amanecer study participants), and a graphical presentation of the main findings (Fig. 1: graphs describing the results in English and in Spanish). For example, the explanation of results for the first RCT of Nuevo Amanecer stated that participants that took part in the program upon enrollment had “better quality of life” and “less discomfort due to their health” compared to the wait group. The graphs for each of the improved outcomes depicted the changes from the start of the study to 6 months, with captions describing how to interpret the results (e.g., “this shows the change in the two groups at the end of the study….”). Aggregate research findings, not individual research results, were returned to the participants. The format and contents of the mailing were consistent across both RCT studies, except that the results provided were specific to each RCT.

The mailing of the results also included a brief one-page questionnaire in plain Spanish and a stamped, self-addressed envelope for return of the completed questionnaire. Community partners reviewed and provided input on the materials that were mailed to the participants. Consistent with a CBPR approach, we returned the aggregate results to the participants and acknowledged the participants’ contributions in a letter.

### Parent RCT studies

The purpose of the two parent RCT studies was to test a new cognitive-behavioral stress management intervention called *Nuevo Amanecer* (A New Dawn), which was developed specifically for Latina breast cancer survivors with extensive community input. The program is available in both English and Spanish and is designed to accommodate persons with limited literacy by using audiovisual supplements and a simple writing style. The program was delivered by peers (trained Spanish-speaking Latina breast cancer survivors) called Compañeras and implemented by community-based organizations (CBOs) who were equal partners in the research. In both the 6-month RCTs, we compared the *Nuevo Amanecer* intervention with a delayed intervention control group (control group participants were offered the program after the trial). Outcome measures consisted of self-report measures of breast cancer-specific quality of life and psychosocial distress.

Community research partners were involved from study inception to dissemination of the results. Details on the CBPR approaches that were used to develop the program and implement the RCTs can be found elsewhere [19–21]. Both trials used identical recruitment and data collection methods; however, there were slight differences in the inclusion criteria. In the first study, eligibility was restricted to women within 1 year since diagnosis while in the second study, this criterion was dropped. The first RCT (*n* = 151) was conducted between 2011 and 2014 in five urban Northern California counties in the San Francisco Bay Area [22]. The second RCT (*n* = 153) was conducted between 2016 and 2018 in rural areas of Imperial, Tulare, and Santa Cruz/Monterey counties [23]. Participants in both trials were Spanish-speaking Latina survivors of non-metastatic breast cancer, tended to be of low socioeconomic status, and mostly of Mexican descent. The CBOs who implemented the study were very experienced in providing health-related services (patient navigation, social services, health education, etc.) in the local Latino community. Lay health workers hired by the CBOs were trained by the research team to conduct recruitment, enrollment, baseline assessments, and randomization for the study. Recruiters identified potential participants through intake records and approached women in person or by telephone to invite women to participate. If the woman was interested and eligible, the recruiter conducted the enrollment, baseline interviews, and randomization procedures in-person, following a standardized protocol.

### Measures

Descriptive characteristics of age in years (30–45/46–55/56–65/65+), educational attainment (6 years or less/more than 6 years to high school/more than high school), employment status (yes/no), experience of financial hardship in the past year (yes/no), and country of birth (Mexico, USA/others) were obtained through the self-reported baseline surveys from the original RCTs. The questionnaire, to be completed after reading the letter and results, asked if the participants read the letter, had problems understanding the results, whether receiving the results influenced their willingness to participate in future studies, and about their preferences for the presentation format of the results. Additional questions included if they had previously received research results, if they felt sharing the results with participants was important, and whether they agreed that receiving the results made them feel important for having participated in the study. A final open-ended question asked if they had any suggestions about how best to provide participants with the results of studies in which they participate.

### Statistical analysis

Descriptive demographic characteristics were used to characterize the sample. The chi-square tests were used to examine the urban/rural differences in demographic characteristics and responses to the structured survey items.

Our main predictors of interest were age, education, and rural/urban residence. Our outcomes were problems understanding the study results (yes vs. no), importance of receiving the results, whether receiving the results made them more willing to participate in future studies (vs. less/likely/did not change), and preference for a graphic (vs. written) format. We used multivariable logistic regression models (with listwise deletion) to test for the independent effects of age, education, and rural/urban residence on each of the outcomes. For these analyses, we recoded responses to the willingness to participate question to create a dichotomous outcome of the following: it made me more likely to participate vs. it made me less likely to participate/did not change how willing I am to participate in the future. Only women who indicated a preference in the format were included in the analysis of the question regarding preferences for graphical vs. written format. All statistical analyses were performed using SAS version 9.4.

## Results

Of the 304 participants (151 urban and 153 rural), 92 returned a completed questionnaire (response rate of 31.3%, not including 10 women who had their letter returned due to an incorrect address or who were deceased). Responders and non-responders did not differ on age (*p* = .38), educational attainment (*p* = .73), employment status (*p* = .33), financial hardship experience in the last year (*p* = .74), country of birth (*p* = .46), urban/rural residence status (*p* = .46), or treatment arm assignment in the original RCT (*p* = .56). The mean age of participants was 54.2 years (SD 11.07), with a majority (66.3%) being between the ages of 46 and 65 years (Table 1). A high proportion of respondents had a high school education or less (80.4%). Overall, 78.3% were unemployed, and 58.2% reported experiencing financial hardship in the past year. There were no significant demographic differences in urban and rural respondents except that women living in rural areas were more likely to be of Mexican origin (81.8% vs. 64.6%, *p* < .001).

**Table 1:** Demographic characteristics of respondents to survey regarding receipt of the results: *Nuevo Amanecer* and *Nuevo Amanecer II* (*N* = 92)

Characteristic	Total, *n* = 92	Urban, *n* = 48	Rural, *n* = 44	*p-*value
Age in years (range 30–88), *n* (%)				0.346
30–45	17 (18.5)	11 (22.9)	6 (13.6)	
46–55	35 (38.0)	20 (41.7)	15 (34.1)	
56–65	26 (28.3)	12 (25.0)	14 (31.8)	
65+	14 (15.2)	5 (10.4)	9 (20.5)	
Educational attainment, *n* (%)				0.513
Elementary (6 years) or less	35 (38.0)	17 (35.4)	18 (40.9)	
More than elementary to HS graduate	39 (42.4)	23 (47.9)	16 (36.4)	
More than HS	18 (19.5)	8 (16.7)	10 (22.7)	
Employed, *n* (%)				0.468
Yes	20 (21.7)	9 (18.8)	11 (25.0)	
No	72 (78.3)	39 (81.3)	33 (75.0)	
Any financial hardship in the past year, *n* (%)				0.191
Yes	53 (5842)	31 (66.0.)	22 (50.0)	
No	38 (41.3)	16 (33.3)	22 (50.0)	
Missing	1 (1.1)	1 (2.1)	0 (0)	
Country of birth, *n* (%)				< 0.001
Mexico	67 (72.8)	31 (64.6)	36 (81.8)	
USA	7 (7.8)	1 (2.1)	6 (13.7)	
Others	18 (19.6)	16 (33.3)	2 (4.6)	

The majority had read the letter describing the results (95.7%) and reported no problems understanding the results of the study (91.1%) (Table 2). Almost all (96.7%) reported they thought sharing the results was very/extremely important (vs. not at all/somewhat important), and they agreed a lot (vs. do not agree/agree a little bit) with the statement that getting the result made them feel as if their participation was very important (92.3%). Regarding the willingness to participate in future research studies, 60.2% of participants felt that receiving the results made them more likely to participate. Concerning the format for returning the results, about half of the participants did not have a preference (51.7%). Most of those reporting a preference indicated they liked written responses better than graphs (37.4% of the total); only 10.9% preferred graphs. There were no differences between urban and rural women on understanding of the results or willingness to participate. Among the subset of women who stated a format preference, rural women preferred the graphs significantly more than urban women (20.9% vs. 2.1%; *p* < .05) (results not tabled).

**Table 2:** Responses of Latina breast cancer survivors toward receiving the study results: *Nuevo Amanecer* and *Nuevo Amanecer II* respondents (*N* = 92)

Question	Total, *n* (%)
1. Did you read the letter describing the results?	
(0) No	0
(1) Yes, all of it	88 (95.7)
(2) Yes, some of it	4 (4.3)
2. Did you have any problems understanding the results of the study?	
(1) No	84 (91.1)
(2) Yes (write in type of problem)	8 (8.7)
3. Before the *Nuevo Amanecer* study, have you ever received the results of a research study that you participated in?	
(0, 2) No (including not been in any other studies)	75 (81.5)
(1) Yes	17 (18.5)
4. How important do you think it is for researchers to share the results with study participants?	
(0–1) Not at all important/somewhat important	3 (3.3)
(2, 3) Very important/extremely important	89 (96.7)
5. How much do you agree with the following statement: getting the results made me feel as if my participation was very important?	
(0) I do not agree.	1 (1.1)
(1) I agree a little bit.	6 (6.6)
(2) I agree a lot.	84 (92.3)
6. Did getting the results of the study change how willing you are to participate in future research studies?	
(1) Yes, it made me more likely to participate in the future.	53 (60.2)
(2) Yes, it made me less likely to participate in the future.	3 (3.4)
(0) No, it did not change how willing I am to participate in the future.	32 (36.4)
7. Which way of describing the results did you like better?	
(1) I liked the written description better.	34 (37.4)
(2) I liked the graphs better.	10 (11.0)
(3) I liked them both the same.	47 (51.7)

For the open-ended question, most of the written comments did not have suggestions regarding the format of the returned results. Instead, most comments were urging researchers to continue working with Latina breast cancer survivors and expressions of appreciation for the study. For example, one woman wrote: “It is really important to continue this program because they (Latina breast cancer survivors) feel really alone during cancer. Don’t leave them alone with this problem.” Some individuals also emphasized the importance of sharing these results with a larger audience to show the value of this type of research.

In the multivariable models adjusted for other variables in the model, among those who stated a format preference (*n* = 44), only residence was related to preference for graphical presentation of results (vs. written), with rural women significantly preferring graphs over a written format (OR = 20.7; 95% CI 1.82, 234.8) (Supplemental Table 1). There were no independent effects of age, education, or residence on problems understanding the results or intent to participate in future studies.

## Discussion

A common concern among ethnic minority communities is that investigators conduct research in their communities and then leave without further communication once the data is collected. Most participants in this study had less than a high school education, did not speak English as their primary language, and experienced financial hardship, Thus, our study sample constituted a very vulnerable group that tends to be underrepresented in research. We aimed to explore the perspectives of Latina breast cancer survivors on their attitudes about and preferences for receiving a summary of simple low-literacy study results. Most women had never participated in research prior to the RCTs for which they received the results. All women reported having read the results, and over 90% reported no problems understanding the results. Almost all reported that it is very/extremely important that researchers share the study results with the participants.

Our findings point to the value of disseminating the results among a vulnerable group of Latina breast cancer survivors. In our project, it was clear that the sample was interested in the results and that sharing the results made them feel that their participation was important, that is, valued by the research team. Whether returning the study results increases the likelihood of participating in future studies among Latina breast cancer survivors and Latinos, in general, remains to be investigated.

Latinos in general tend to express a willingness to participate in research [24] and are more likely to be willing to participate in cancer intervention studies than Whites [25, 26]. Empirical data on the return of results among Latinos is lacking. Our results are consistent with those from two behavioral studies that could be found that focused on return of results and were conducted among vulnerable Latino participants. In both studies, disseminating aggregate results was associated with more favorable attitudes toward research participation [27, 28]. Disseminating research findings to the participants and the community is critical and consistently overlooked, even in studies using participatory approaches.

As suggested by community partners in our studies, consideration of the literacy level of participants when disseminating the results is important. Taking this into account, the materials were developed and presented in an easy-to-understand format using both plain language and graphs. The majority of respondents reported reading the results and no trouble understanding them. We found that among women who stated a preference in format, rural women were more likely to prefer graphical results, compared to urban women, independent of age and education. This could be due to a variety of factors, including difficulty in understanding written results and limited literacy.

The majority of women reported not having participated in research projects before the Nuevo Amanecer study, but they believed that receiving the results was “very/extremely important.” They also indicated that their participation in the research “made them feel important.” This suggests that returning the results to the participants is crucial for allowing Latina breast cancer survivors to feel valued and recognized for their significant contributions. It also builds a sense of community in research, as they feel their participation can help other women who are going through the same experience. The most common theme identified in the responses to the open-ended question asking for suggestions on how to return the results was not related to the topic, but was instead an expression of immense gratitude for the opportunity to participate in the RCT projects and a plea for more research focused on Latina cancer survivors. Continued research with Latina breast cancer survivors fills a critical need in this understudied population.

More research that systematically tests various methods for aggregate return of results are clearly warranted. Our descriptive cross-sectional study represents a first step and is novel in that it examines participant experiences and opinions with research among especially vulnerable women, that is, Spanish-speaking Latina breast cancer survivors, including those from both urban and rural communities. Our study demonstrates that women responding to our survey read and understood the results and believe strongly that research participants should be provided with the study findings. As a descriptive study in a critically underrepresented population in research, this study is an important first step that draws attention to a neglected area, return of results to the study participants.

### Limitations

Our low response rate may have introduced bias and limits the external validity of our findings; women who responded to the survey may have been more inclined to participate in research than non-responders. Follow-up attempts to secure the completed questionnaire were limited by insufficient resources for this project. In future studies, increased follow-up attempts would be made as well as use of randomized study designs to empirically test various methods and formats for return of results in specific populations. Only the variables of age, education, and rural/urban residence were included in the models; we did not control for additional potential confounders and did not adjust for multiple comparisons. The outcome of willingness (intent) to participate in future studies may not accurately predict future behavior, and socially desirable and acquiescence response biases may have been present. It is possible that only women who understood the results responded to the survey. Randomized studies comparing a group who receives the results with a group who does not to evaluate prospectively the effects on intent and behavior relative to participating in research can help address some of these limitations. Finally, our findings may not generalize to other Latino samples, since our study participants were predominantly Spanish-speaking low-income Latinas who had a high school education or less.

## Conclusions

Lessons learned include that sharing research results with our participants in plain language yields few problems in understanding the results, makes participants feel appreciated, and is highly valued by vulnerable Latina breast cancer survivors. The small body of research on dissemination of aggregate results in behavioral studies suggests that these lessons apply broadly to behavioral research conducted in communities experiencing social and structural disadvantage. Returning the results and understanding the impact of doing so is consistent with maintaining community involvement in all phases of research and is only one step in methods for enhancing resources and power in vulnerable communities [28]. A consistent practice among researchers of returning the results could help to counter common barriers to participation in research among minority communities, including feelings of mistrust and views that most researchers perform “helicopter” research where they never return to the communities once they have the data. This study highlights the need for health disparity researchers to engage communities throughout all stages of research, including the dissemination stage. Doing so conveys that participants’ contributions are valued by the researchers and is consistent with participants’ desire and appreciation for receiving the results.

## Supplementary Information

**Additional file 1: Figure S1.** English and Spanish versions of letter with aggregate RCT results sent to Nuevo Amanecer study participants.

**Additional file 2: Table S1.** Problems understanding study results, willingness to participate in future studies, and preference for format, Nuevo Amanecer RCT Studies (*n*=92).

## Data Availability

All materials and datasets are available from the corresponding author upon request.

## Abbreviations

CBPR: Community-based participatory research
RCTs: Randomized controlled trials
COVID-19: Coronavirus disease 19

## References

[1] Cortés YI, Arcia A, Kearney J, Luchsinger J, Lucero RJ (2017). Urban-dwelling community members’ views on biomedical research engagement. Qual Health Res..

[2] Scharff DP, Mathews KJ, Jackson P, Hoffsuemmer J, Martin E, Edwards D (2010). More than Tuskegee: understanding mistrust about research participation. J Health Care Poor Underserved..

[3] Flicker S, Travers R, Guta A, McDonald S, Meagher A (2007). Ethical dilemmas in community-based participatory research: recommendations for institutional review boards. J Urban Health..

[4] Chastain D, Osae S, Henao A, Franco-Paredes C, Chastain J, Young H (2020). Racial disproportionality in COVID clinical trials. N Engl J Med..

[5] Lackland DT, Sims-Robinson C, Jones Buie JN, Voeks JH (2020). Impact of COVID-19 on clinical research and inclusion of diverse populations. Ethn Dis..

[6] Ratneswaren A (2020). The I in COVID: the importance of community and patient involvement in COVID-19 research. Clin Med..

[7] Collins SE, Clifasefi SL, Stanton J, KJE S, Gil-Kashiwabara E, Rodriguez Espinosa P, Nicasio AV, Andrasik MP, Hawes SM, Miller KA, Nelson LA, Orfaly VE, Duran BM, Wallerstein N, The Leap Advisory B (2018). Community-based participatory research (CBPR): towards equitable involvement of community in psychology research. Am Psychol..

[8] Purvis RS, Abraham TH, Long CR, Stewart MK, Warmack TS, McElfish PA (2017). Qualitative study of participants’ perceptions and preferences regarding research dissemination. AJOB Empir Bioeth..

[9] Shalowitz DI, Miller FG (2008). The search for clarity in communicating research results to study participants. J Med Ethics.

[10] Wilkins CH, Mapes BM, Jerome RN, Villalta-Gil V, Pulley JM, Harris PA (2019). Understanding what information is valued by research participants, and why. Health Aff..

[11] Miller FA, Christensen R, Giacomini M, Robert JS (2008). Duty to disclose what? Querying the putative obligation to return research results to participants. J Med Ethics..

[12] Partridge AH, Wolff AC, Marcom PK, Kaufman PA, Zhang L, Gelman R, Moore C, Lake D, Fleming GF, Rugo HS, Atkins J, Sampson E, Collyar D, Winer EP (2009). The impact of sharing results of a randomized breast cancer clinical trial with study participants. Breast Cancer Res Treat..

[13] Beskow LM, Burke W, Fullerton SM, Sharp RR (2012). Offering aggregate results to participants in genomic research: opportunities and challenges. Genet Med..

[14] Kerasidou A (2015). Sharing the knowledge: sharing aggregate genomic findings with research participants in developing countries. Dev World Bioeth..

[15] Wong CA, Hernandez AF, Califf RM (2018). Providing individual research results to participants-reply. JAMA..

[16] Levac L, Ronis S, Cowper-Smith Y, Vaccarino O (2019). A scoping review: the utility of participatory research approaches in psychology. J Community Psychol..

[17] Long CR, Stewart MK, McElfish PA (2017). Health research participants are not receiving research results: a collaborative solution is needed. Trials..

[18] Chen PG, Diaz N, Lucas G, Rosenthal MS (2010). Dissemination of results in community-based participatory research. Am J Prev Med..

[19] Napoles AM, Santoyo-Olsson J, Ortiz C, Gregorich S, Lee HE, Duron Y, Graves K, Luce JA, McGuire P, Diaz-Mendez M, Stewart AL (2014). Randomized controlled trial of Nuevo Amanecer: a peer-delivered stress management intervention for Spanish-speaking Latinas with breast cancer. Clin Trials..

[20] Napoles AM, Santoyo-Olsson J, Stewart AL, Ortiz C, Garcia-Jimenez M (2018). Evaluating the implementation of a translational peer-delivered stress management program for Spanish-Speaking Latina breast cancer survivors. J Cancer Educ..

[21] Santoyo-Olsson J, Stewart AL, Samayoa C, Palomino H, Urias A, Gonzalez N, Torres-Nguyen A, Coleman L, Escalera C, Totten VY, Ortiz C, Napoles AM (2019). Translating a stress management intervention for rural Latina breast cancer survivors: the Nuevo Amanecer-II. PLoS One..

[22] Napoles AM, Ortiz C, Santoyo-Olsson J, Stewart AL, Gregorich S, Lee HE, Duron Y, McGuire P, Luce J (2015). Nuevo Amanecer: results of a randomized controlled trial of a community-based, peer-delivered stress management intervention to improve quality of life in Latinas with breast cancer. Am J Public Health..

[23] Napoles AM, Santoyo-Olsson J, Stewart AL, Ortiz C, Samayoa C, Torres-Nguyen A, Palomino H, Coleman L, Urias A, Gonzalez N, Cervantes SA, Totten VY (2020). Nuevo Amanecer-II: results of a randomized controlled trial of a community-based participatory, peer-delivered stress management intervention for rural Latina breast cancer survivors. Psychooncology..

[24] Garza MA, Quinn SC, Li Y, Assini-Meytin L, Casper ET, Fryer CS, Butler J, Brown NA, Kim KH, Thomas SB (2017). The influence of race and ethnicity on becoming a human subject: factors associated with participation in research. Contemp Clin Trials Commun..

[25] Echeverri M, Anderson D, Napoles AM, Haas JM, Johnson ME, Serrano FSA (2018). Cancer health literacy and willingness to participate in cancer research and donate bio-specimens. Int J Environ Res Public Health.

[26] Wendler D, Kington R, Madans J, Van Wye G, Christ-Schmidt H, Pratt LA, Brawley OW, Gross CP, Emanuel E (2006). Are racial and ethnic minorities less willing to participate in health research?. PLoS Med..

[27] Heerman WJ, Wilkins CH, Barkin SL (2021). Disseminating aggregate research findings to participants. Pediatr Res..

[28] Flaskerud JH, Anderson N (1999). Disseminating the results of participant-focused research. J Transcult Nurs..

