# Supplementary figures and images for: Sharing research results with Latina breast cancer survivors who participated in a community-engaged behavioral RCT study: a descriptive cross-sectional survey study
Source: Trials. 2022 Jan 8;23:25. doi: 10.1186/s13063-021-05945-8 (PMC8742155; doi:10.1186/s13063-021-05945-8)

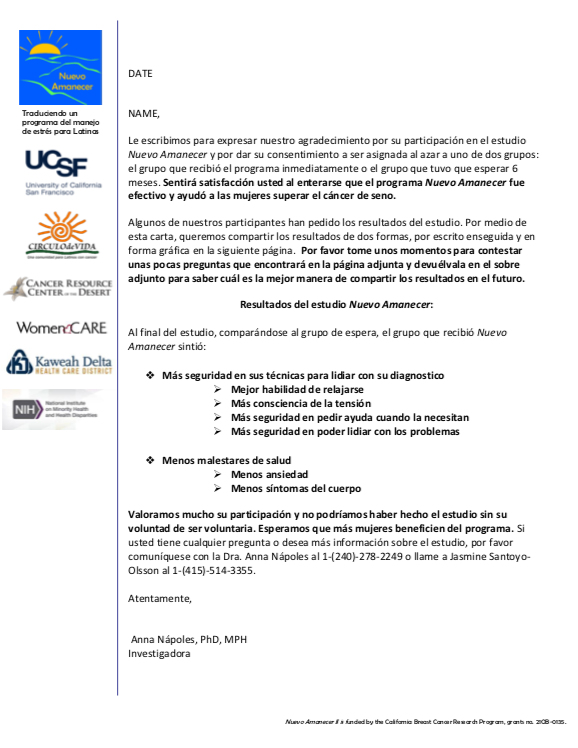

Supplement: Supplementary file 1 — Additional file 1: Figure S1. English and Spanish versions of letter with aggregate RCT results sent to Nuevo Amanecer study participants. [file 13063_2021_5945_MOESM1_ESM.docx]
