# Supplementary material for: Sharing research results with Latina breast cancer survivors who participated in a community-engaged behavioral RCT study: a descriptive cross-sectional survey study
Source: Trials. 2022 Jan 8;23:25. doi: 10.1186/s13063-021-05945-8 (PMC8742155; doi:10.1186/s13063-021-05945-8)
Supplement: Supplementary file 2 — Additional file 2: Table S1. Problems understanding study results, willingness to participate in future studies, and preference for format, Nuevo Amanecer RCT Studies (n=92). [file 13063_2021_5945_MOESM2_ESM.docx]

|  | Problems understanding study results^a^ | | Willingness to participate in future studies^b^ | | Preference for graphic or written format^c^ | |
| --- | --- | --- | --- | --- | --- | --- |
|  | AOR^d^ | p-value | AOR^d^ | p-value | AOR^d^ | p-value |
| Age in years |  |  |  |  |  |  |
| 30-45 | REF |  | REF |  | REF |  |
| 46-55 | 0.401 | 0.426 | 1.249 | 0.7466 | 0.620 | 0.664 |
| 56-65 | 0.746 | 0.892 | 1.112 | 0.9911 | 0.563 | 0.592 |
| 65+ | 0.727 | 0.943 | 1.120 | 0.9952 | 1.641 | 0.510 |
| Educational attainment |  |  |  |  |  |  |
| More than high school | REF |  | REF |  | REF |  |
| More than elementary to high school graduate | 1.481 | 0.215 | 1.104 | 0.7476 | 0.207 | 0.606 |
| Elementary (6 years) or less | 0.261 | 0.168 | 1.637 | 0.3476 | 0.125 | 0.189 |
| Urbanicity |  |  |  |  |  |  |
| Urban | REF |  | REF |  | REF |  |
| Rural | 2.235 | 0.322 | 1.367 | 0.4867 | 20.714 | 0.014 |

^a^ “Did you have any problem understanding the results of the study?”; response options: yes or no.

^b^ “Did getting the results of the study change how willing you are to participate in future research studies?” Response options were combined as: more willing to participate vs less likely to participate/did not change how willing they were (reference).

^c^ “Which way of describing the results did you like better?” Responses were: graphic vs written format (reference); only incudes those who indicated a preference (n=44).

^d^ AOR: Adjusted odds ratio, adjusted for other variables in the model.

Supplemental Table 1: Problems understanding study results, willingness to participate in future studies, and preference for format, *Nuevo Amanecer* RCT Studies (n=92)
